# Investigating the Formation of Polymer–Nanoparticle Complex Coacervate Hydrogels Using Polymerization-Induced Self-Assembly-Derived Nanogels with a Succinate-Functional Core

**DOI:** 10.1021/acs.langmuir.4c02626

**Published:** 2024-09-18

**Authors:** Ruiling Du, Xueyuan Li, Lee A. Fielding

**Affiliations:** † Department of Materials, School of Natural Sciences, University of Manchester, Oxford Road, Manchester M13 9PL, U.K.; ‡ Henry Royce Institute, The University of Manchester, Oxford Road, Manchester M13 9PL, U.K.

## Abstract

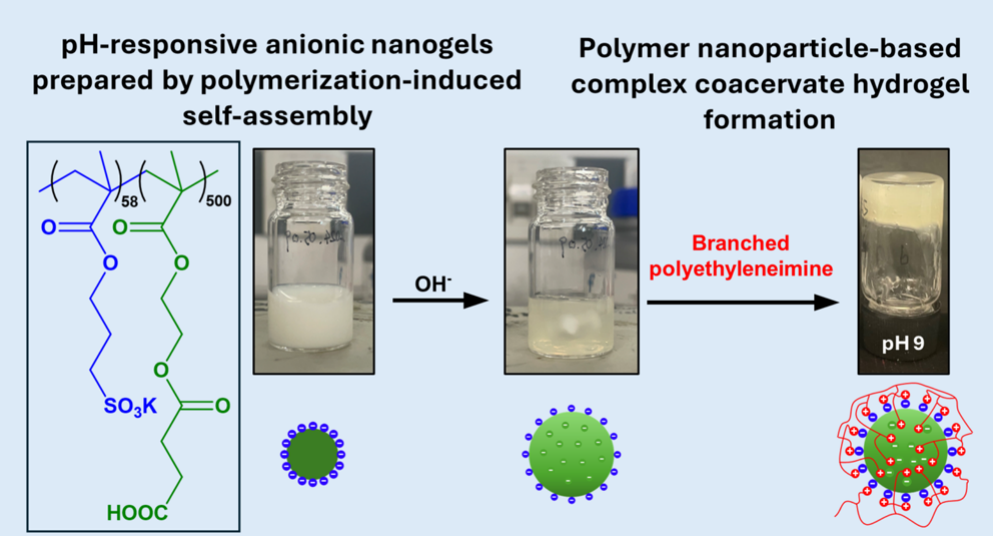

This paper reports polymer–nanoparticle-based complex coacervate (PNCC) hydrogels prepared by mixing anionic nanogels synthesized by polymerization-induced self-assembly (PISA) and cationic branched poly(ethylenimine) (bPEI). Specifically, poly(3-sulfopropyl methacrylate)_58_-*b*-poly(2-(methacryloyloxy)ethyl succinate)_500_ (PKSPMA_58_-PMES_500_) nanogels were prepared by reversible addition–fragmentation chain-transfer (RAFT)-mediated PISA. These nanogels swell on increasing the solution pH and form free-standing hydrogels at 20% w/w and pH ≥ 7.5. However, the addition of bPEI significantly improves the gel properties through the formation of PNCCs. Diluted bPEI/nanoparticle mixtures were analyzed by dynamic light scattering (DLS) and aqueous electrophoresis to examine the mechanism of PNCC formation. The influence of pH and the bPEI-to-nanogel mass ratio (MR) on the formation of these PNCC hydrogels was subsequently investigated. A maximum gel strength of 1300 Pa was obtained for 20% w/w bPEI/PKSPMA_58_-PMES_500_ PNCC hydrogels prepared at pH 9 with an MR of 0.1, and shear-thinning behavior was observed in all cases. After the removal of shear, these PNCC gels recovered rapidly, with the recovery efficiency being pH-dependent.

## Introduction

Complex coacervation refers to the phenomenon of the formation of separate phases in one solvent with a clear interfacial boundary, caused by the interaction of two oppositely charged macromolecules in a homogeneous colloidal solution.^1^ This process has been widely used as a strategy for encapsulation in cosmetic,^2−4^ pharmaceutical,^5−7^ and food science industries.^8−10^ It is noteworthy that mixing two aqueous solutions of oppositely charged polyelectrolytes generally leads to phase separation.^11,12^ However, judicious modification of the synthetic polymer can effectively prevent macroscopic phase separation and produce a new class of physical hydrogels made from polyelectrolyte-regulated coacervate assembly, yielding an interesting range of soft materials.^13−21^ Similar to the majority of other physical hydrogels, the gelation process of coacervates is relatively easy to achieve and is easy to reverse when necessary.

Among the many known cationic polyelectrolytes, branched poly(ethylenimine) (bPEI) is one of the most studied polycations due to its high nucleophilicity, thermal stability, and eco-friendly characteristics.^22^ Branched PEI is a weak cationic polyelectrolyte containing abundant amine groups. Since every third atom of bPEI is a protonatable amino nitrogen atom, it can serve as an effective ionic branching point over a wide pH range.^23^ Polyelectrolyte complexes comprising bPEI have been prepared across differing length scales to generate materials ranging from nanoparticles (NPs)^24,25^ to films^26^ and macroscopic gels.^21,27^

It was reported that the adsorption behavior of bPEI onto anionic polyelectrolyte surfaces via electrostatic interaction occurs in three conformations: trains, loops, and tails.^28^ This is an important factor in determining the macroscopic properties of mixed polyelectrolytes containing bPEI. Zhang and co-workers^29^ reported a liquid–gel–liquid transition in mixed suspensions of a silica colloid and bPEI, demonstrating that bPEI molecules act as bridges between silica particles. The amount and conformation of bPEI bridges can self-arrange with different solution environments, such as pH and ionic strength, which determine the performance of this colloid/polyelectrolyte mixed system.

Wu and co-workers^21^ explored a polymer/microgel complex coacervate (PMCC) hydrogel formed by mixing preformed polyacid microgels and cationic bPEI in an aqueous solvent. The coassembly of the polymer/microgel polyelectrolytes formed a hydrogel that was shapeable, superstretchable, self-healing, and adhesive. In addition, these materials exhibited low cytotoxicity and could be toughened using Ca^2+^. More recently, this new family of hydrogels have been enhanced with graphene nanoplatelets, which endowed them with electrical conductivity and the ability to sense tensile and compressive stains.^30^ However, such microgels were synthesized by using seed-feed emulsion polymerization. Although this method allows for the production of particles with tunable sizes and swelling ratios, it is relatively limited in its capacity to produce NPs with precise molecular weight, functionality distribution, and morphological control.

Polymerization-induced self-assembly (PISA) is a powerful and versatile platform technology for the rational synthesis of sterically stabilized diblock copolymer NPs of controllable size, shape, and functionality.^31−34^ Since reversible addition–fragmentation chain transfer (RAFT)-mediated PISA has broad applicability to a range of monomers, this technology offers a versatile platform for designing various nanoparticles.^35−37^ Inspired by the structure of PMCC hydrogels, PISA-derived spherical nanoparticles with anionic functional groups are potentially able to interact with cationic bPEI to generate polymer–nanoparticle complex coacervate (PNCC) hydrogels. In previous work,^38^ our group investigated the preparation of PNCC hydrogels via mixing PISA-derived poly(3-sulfopropyl methacrylate)-*b*-poly(methacrylic acid-*stat*-benzyl methacrylate) (PKSPMA-P(BzMA-*stat*-MAA)) NPs and bPEI at various bPEI-to-NP mass ratios (MR). It was demonstrated that the incorporation of MAA within the core-forming block of the NPs imparted pH-responsive nanogel behavior and was essential for subsequent PNCC hydrogel formation.

While PISA-derived nanogels with thermoresponsive behavior have been widely reported,^39−41^ there are relatively limited examples where the core of the NPs provides pH-responsive functionality.^38,42^ Recently, we developed a new generation of nanogels prepared via RAFT-mediated emulsion PISA with a single monomer as the pH-responsive core-forming block.^42^ Specifically, PKSPMA macromolecular chain transfer agents (macro-CTAs) were block-extended with carboxylic acid-functional 2-(methacryloyloxy)ethyl succinate (MES) to generate well-defined pH-responsive nanogels that swell in basic conditions. Notably, these nanogels were successfully formed even without the addition of an additional cross-linking comonomer.^42^

Herein, binary polyelectrolyte combinations of the recently developed PISA-derived anionic PKSPMA_58_-PMES_500_ nanogels and cationic bPEI are investigated to demonstrate the relative simplicity and versatility for this method of PNCC hydrogel preparation. The chosen nanogels were prepared with a PKSPMA_58_ macro-CTA using RAFT aqueous emulsion polymerization of MES at pH 2 and 20% w/w. The resulting size, morphology, and swelling behavior of the NPs were characterized via transmission electron microscopy (TEM), dynamic light scattering (DLS), and aqueous electrophoresis. Subsequently, a series of bPEI/NP PNCCs were prepared at different pHs via mixing NPs and bPEI while varying the bPEI-to-NP MR, and the rheological properties were investigated.

## Experimental Section

### Materials

**Figure 1:**
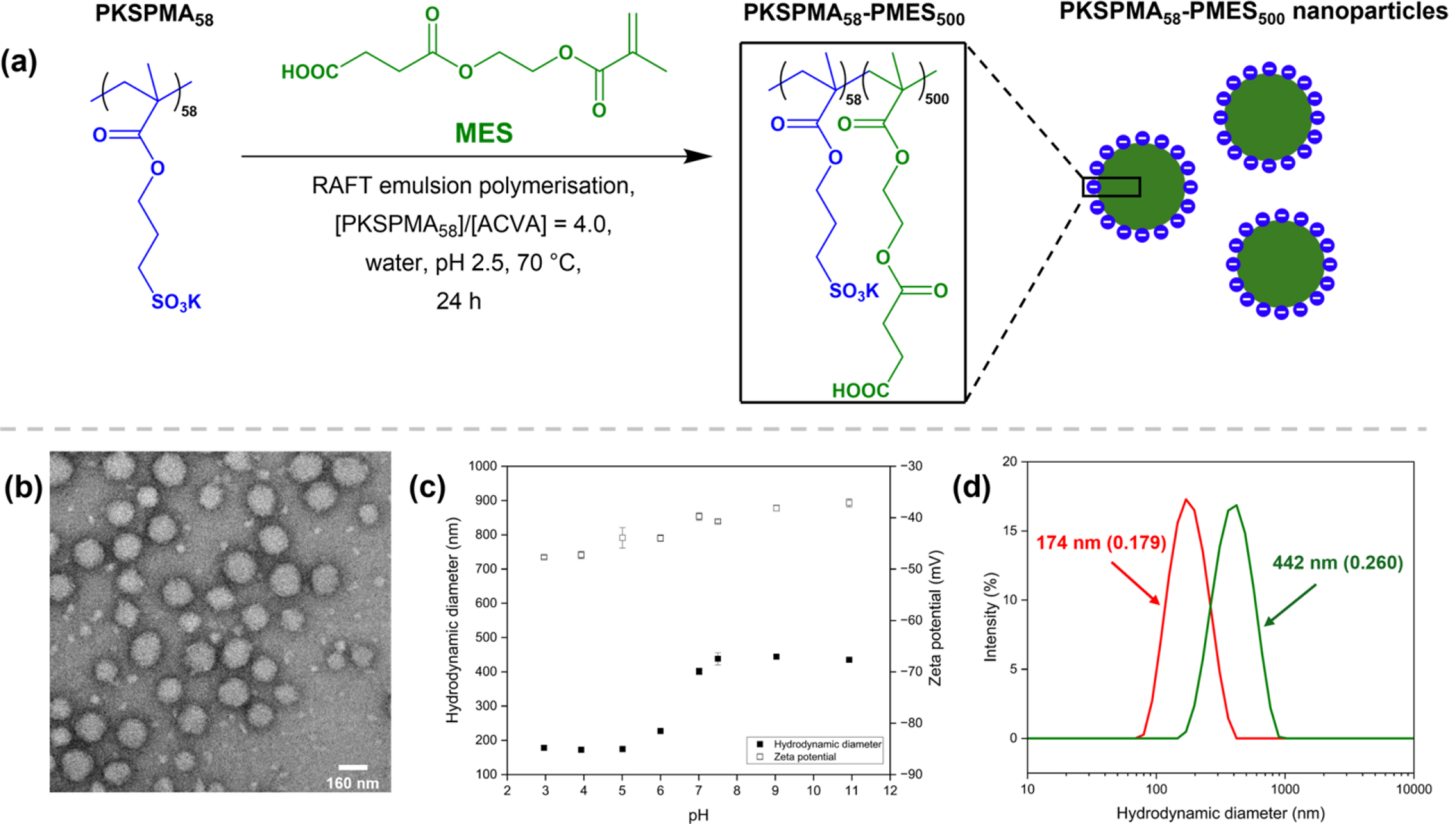
(a) Synthesis of PKSPMA_58_-PMES_500_ nanoparticles via the RAFT-mediated polymerization of MES in water at 70 °C. (b) Representative TEM image. (c) Corresponding DLS and aqueous electrophoresis data as a function of pH. (d) DLS intensity-average size distributions at pH 3 and pH 9 (25 °C, 0.10% w/w, 1 mM KCl; the number in parentheses represents the DLS polydispersity index).

Branched poly(ethylenimine) (bPEI) aqueous solution (50 wt %, *M*_n_ 60 kDa) was purchased from Sigma-Aldrich (U.K.) and used as received. Potassium hydroxide (KOH), potassium chloride (KCl), and hydrochloric acid (HCl, ∼37%) were purchased from Fisher Scientific (U.K.) and diluted in-house. Deionized water was obtained from an in-house SUEZ Purite Analyst water purification unit. PKSPMA_58_ macro-CTA (Figures S1 and S2) and PKSPMA_58_-PMES_500_ NPs (Figure 1) were prepared in-house following previously published protocols, which are described in the Supporting Information.^38,42,43^

### Protocol for Preparing 20% w/w bPEI Solutions

bPEI was diluted to 20% (w/w) using aqueous solutions of HCl prepared at different concentrations to achieve the desired pH. Figure S3 summarizes the pH obtained for 20% (w/w) bPEI solutions diluted with different concentrations of HCl. The following example describes the preparation of a 20% w/w bPEI solution at pH 7.5 via mixing bPEI solution (20% w/w, pH 8.1) and bPEI solution (20% w/w, pH 6.2). 4 g of 50 wt % bPEI solution was transferred to a 14 mL vial, and then 6 g of 3.5 M HCl was added into the 14 mL vial. This mixture was vortexed vigorously for 30 s at room temperature and left in a sealed vial for 10 min before measuring the pH of the diluted bPEI solution (20% w/w), which in this case was pH 8.1. Similarly, bPEI solution (50 wt %, 4 g) was mixed with 5 M HCl (6 g) resulting in a diluted bPEI solution at pH 6.2 (20% w/w). Therefore, bPEI solution (20% w/w, pH 7.5) was obtained by gradually adding bPEI solution (20% w/w, pH 6.2) to bPEI solution (20% w/w, pH 8.1) until the pH of the 20% w/w bPEI solution reached pH 7.5. The pH of all samples was measured using a Mettler Toledo FiveEasy bench meter equipped with a Hannah HI-1330B glass body refillable pH semi-micro-electrode probe.

### Preparation of bPEI/NP PNCCs

Each sample herein is denoted using the following format: bPEI/NP (pH-MR), where pH is the pH of both the NP dispersion and the bPEI solution and MR corresponds to the bPEI-to-NP mass ratio. The following example describes the preparation of bPEI/NP (9–0.1). PKSPMA_58_-PMES_500_ nanogel dispersion (2 g, 20% w/w) was transferred to a 7 mL vial. Then, the pH of the nanogel dispersion was increased to 9 by adding a saturated KOH solution (0.14 g). 0.2 g of bPEI solution (20% w/w, pH 9) was injected into the nanogel dispersion. The mixture was mechanically stirred by using a spatula for 3 min and then left in the sealed vial at room temperature for at least 24 h prior to rheology analysis.

### Dynamic Light Scattering and Aqueous Electrophoresis

Dynamic light scattering (DLS) studies were conducted using a Malvern Zetasizer Ultra instrument to measure both the hydrodynamic diameter (*D*_h_) and the ζ-potential. The instrument was equipped with a He–Ne solid-state laser operating at 633 nm, detecting backscattered light at a scattering angle of 173°. Both nanogel dispersions and bPEI solutions were diluted to 0.1% w/w using 1.0 mM KCl as a background electrolyte, and the pH of each diluted sample was adjusted using KOH (2.5/0.25 M) and HCl (2.5/0.25 M). Samples were prepared by mixing diluted nanogel dispersions and diluted bPEI solutions of the same pH value at different MRs. All DLS samples were equilibrated for 240 s before measurements were taken and data were averaged over three consecutive runs. Disposable folded capillary cells (Malvern DTS1070) were used for measuring both the hydrodynamic diameter (*D*_h_) and the ζ-potential.

### Transmission Electron Microscopy

Transmission electron microscopy (TEM) observations were carried out on an FEI Tecnai G2 F20 instrument operating at an accelerating voltage of 200 kV and connected to a Gatan 1k CCD camera. Copper/palladium TEM grids (Agar Scientific, U.K.) were surface-coated with a thin film of amorphous carbon and then subjected to a plasma glow discharge for 30 s to produce a hydrophilic surface. The obtained dispersions were diluted from 20% w/w to 0.1% w/w solids, and a hydrophilic grid was placed onto an aqueous droplet (40 μL) of diluted dispersion for 1 min and then blotted with filter paper to remove excess solution. This grid was then negatively stained by placing onto a uranyl acetate droplet (0.5% w/w, 40 μL) for 30 s. Excess stain was removed by blotting, and the grid was carefully dried with a vacuum hose.

### Rheology Measurements

Rheological measurements were performed on a HAAKE MARS iQ Air rheometer (Thermo Scientific Instruments) equipped with a Peltier stage. A 35 mm, 2° cone geometry was used for all experiments. Storage modulus (*G*′) and loss modulus (*G*″) as a function of strain were measured between 0.03 and 2000% at 25 °C via dynamic oscillatory strain amplitude sweep mode at a frequency of 10 rad s^–1^. The recovery of material properties following network rupture at high strains was investigated via step strain measurements. A high-magnitude strain (ε = 500%) was applied to break the hydrogel structure, followed by a low-magnitude strain (ε = 0.5%) to monitor the rate and extent of recovery of the bulk properties. RheoWin 4 software was used to analyze all of the rheology data.

## Results and Discussion

### Effect of pH on PKSPMA_58_-PMES_500_ Nanoparticles and bPEI

In previous work,^42^ PKSPMA-PMES nanogels were prepared via RAFT-mediated PISA using PKSPMA as a macro-CTA. Briefly, PKSPMA-PMES diblock copolymer nanogels with controllable particle diameters and different swelling ratios were obtained on a relatively small scale via RAFT emulsion polymerization at pH 2 by target varying degrees of polymerization (DP) of the core-forming PMES block. Herein, a scaled-up synthesis (∼25 mL) was conducted to form PKSPMA-PMES particles using a PKSPMA_58_ stabilizer (Figures S1 and S2) and a core-forming PMES block with a target DP of 500 (Figure 1a). The target DP of PMES at 500 nm was chosen to investigate the preparation of PNCCs because NPs with a PMES_500_ core are relatively large (175 nm, Figure 1b) and have a relatively high swelling ratio (Figure 1d). The response of the PKSPMA_58_-PMES_500_ NPs as a function of pH was analyzed by titrating the nanoparticle dispersions and monitoring their mean hydrodynamic diameter and ζ-potential (Figure 1c). On increasing the pH, the NPs become swollen (to approximately 440 nm) and, perhaps surprisingly, do not dissolve. This was previously attributed to the possibility of *in situ* core-cross-linking taking place during RAFT emulsion polymerization.^42^ Nanogels with higher swelling ratios may be able to form hydrogels with improved gel properties due to their higher water capacity while swollen. It is noteworthy that the volume swelling ratio (*D*_h,pH9_/*D*_h,pH3_) of the PKSPMA_68_-P(BzMA_0.6_-*stat*-MAA_0.4_)_300_ nanogels reported in our previous PNCC study was 1.4,^38^ while the volume swelling ratio of PKSPMA_58_-PMES_500_ particles used herein was higher, at 2.5.

The ζ-potential for PKSPMA_58_-PMES_500_ NPs remained highly negative (<−30 mV) at all pH values due to the presence of the highly anionic PKSPMA stabilizer (Figure 1c). Interestingly, the ζ-potential value increases slightly on the pH increasing from 5 to 7, although more carboxylic acid groups in the NP core will have become deprotonated. This is because electrophoretic mobility measurements are only sensitive to charge within a distance of approximately the electrical double layer of the particle surface, and as the swelling of the core increased, the charge density of the KSPMA groups in the shell decreased due to the larger particle volume and so the magnitude of the recorded ζ-potential decreased.

For bPEI, the degree of protonation can be adjusted by varying the solution pH, increasing gradually from 0 at pH 12 to 1.0 at pH 2.^29^ Values for the degree of protonation of bPEI reported in the literature are 0.71, 0.22, and 0.01 at pHs 3, 7, and 10, respectively.^44^ The degree of ionization of the PMES_500_ core was determined using eq 1:
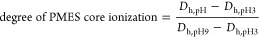
1where *D*_h,pH3_, *D*_h,pH9_, and *D*_h,pH_ are the mean hydrodynamic diameters of PKSPMA_58_-PMES_500_ NPs at pH 3, pH 9, and the specific pH, respectively.

The calculated degree of ionization for 20% w/w PKSPMA_58_-PMES_500_ dispersions and the previously reported degree of protonation for 20% w/w bPEI solutions are plotted in Figure S4. According to Figure S4, four pH values (pH 3, 7.5, 9, and 11) were selected for investigations into binary mixtures of PKSPMA_58_-PMES_500_ NP dispersions and bPEI solutions. These were chosen because at pH 3, the NPs are unswollen and bPEI has a relatively high charge density; at pH 7.5 and 9, the NPs are swollen and bPEI has a relatively low charge density; and at pH 11, the NPs are swollen and bPEI has relatively low charge density.

### DLS Studies on Diluted bPEI/NP Mixtures

**Figure 2:**
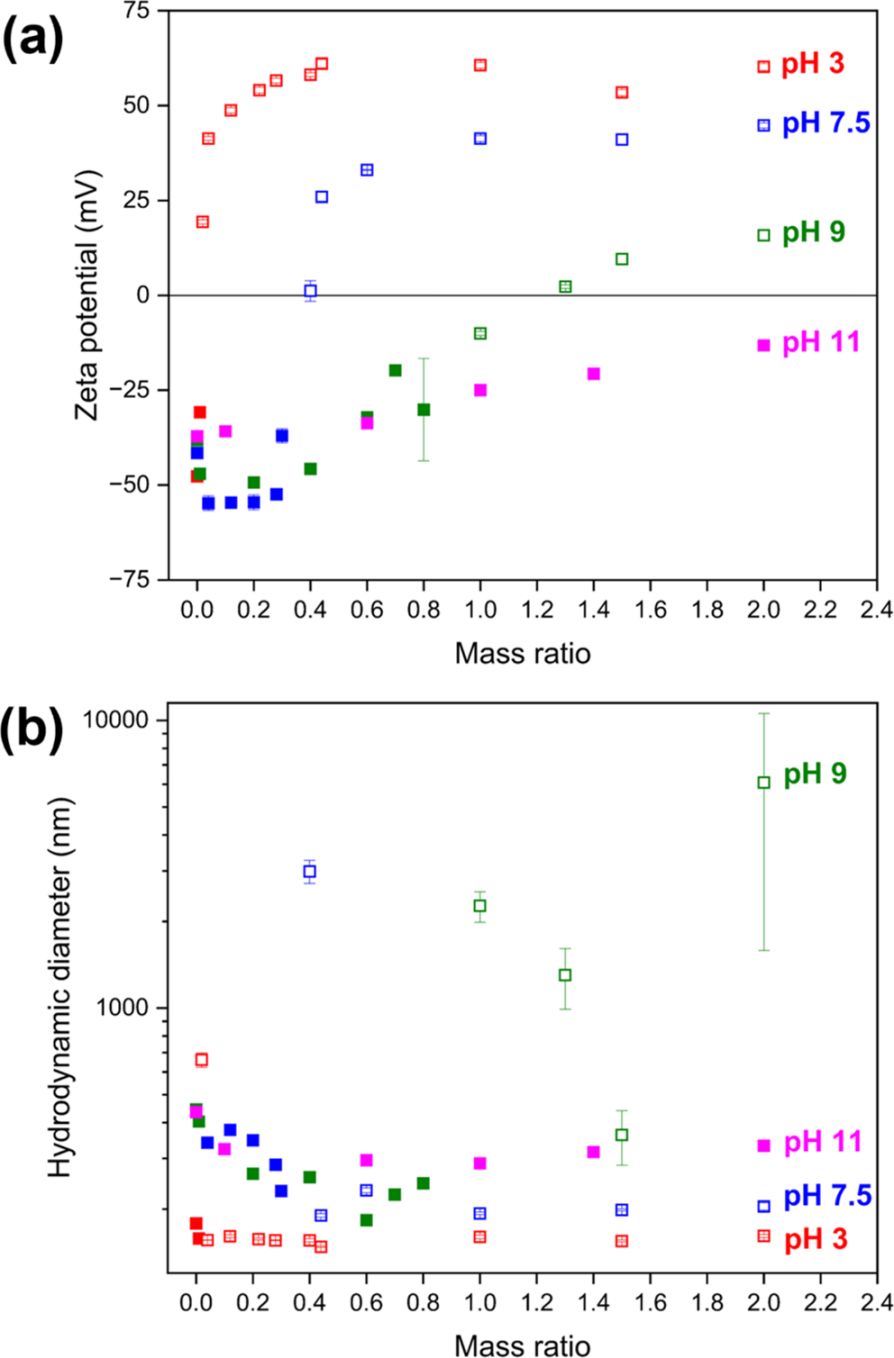
(a) Aqueous electrophoresis data and (b) mean hydrodynamic diameter as a function of pH for dilute aqueous dispersions containing bPEI/NP mixtures at different pH values (pH 3, red; pH 7.5, blue; pH 9, green; and pH 11, pink) with various bPEI-to-NP MRs. Homogeneous samples are represented as solid squares, and phase-separated samples are shown as open squares. Measurements were conducted at a copolymer concentration of approximately 0.1% (w/w) in the presence of 1 mM KCl as a background electrolyte.

The interactions between bPEI and PKSPMA_58_-PMES_500_ NPs were investigated by DLS and aqueous electrophoresis measurements as a function of the mass ratio. Specifically, dilute aqueous PKSPMA_58_-PMES_500_ NP dispersions (0.1% w/w) and bPEI solutions (0.1% w/w) with the same pH were mixed at different bPEI-to-NP MRs; see Figure 2a,2b.

At pH 3, the measured ζ-potential increased sharply with the addition of a small amount of bPEI, transforming from anionic (−31 mV at MR 0.01) to cationic (+19 mV at MR 0.02). This was due to the high density of cationic amine groups within bPEI at low pH only needing to balance the sulfonate groups in the NP coronas. Thus, relatively small amounts of bPEI could compensate the negatively charged NPs and coat the particles tightly in a train conformation,^45^ leading to flocculation at MR 0.02 (Figure 2b, red points). At MR > 0.02, aggregates formed and sedimented, but residual individual particles were nevertheless still detected with positive surface charges (>40 mV).

At pH 7.5, the measured ζ-potential remained highly negative (<−35 mV) from MR 0 to 0.30 and a sharp increase in the ζ-potential started above this value. Ionization of the PMES_500_ cores at pH 7.5 generated more negative groups in the NPs and the charge density of bPEI at pH 7.5 is reduced when compared to pH 3 (Figure S4). Therefore, these anionic NPs required more bPEI to be added (MR = 0.40) to balance their overall charge (Figure 2a, blue data). At MRs ≤ 0.30, the amount of bPEI was not enough to compensate the anionic charge on the PKSPMA_58_-PMES_500_ NPs and thus formed stable swollen nanogels with an adsorbed layer of bPEI. At MR 0.40, the ζ-potential value approached zero, indicating the anionic charges on the NP surfaces were neutralized by bPEI, and no longer able to stabilize these NPs. Thus, flocculation occurred above this MR (Figure 2b, blue data; phase-separated samples are shown as open squares in this figure). Similar to the phenomenon observed for DLS samples prepared at pH 3, at MR > 0.40, sediments formed but individual particles were still detected with a positive surface charge (>25 mV).

It is noteworthy that at pH 3 and pH 7.5, when the MR was higher than the MR at which flocculation occurred, the reported DLS particle size decreased back to relatively small values of approximately 150 and 200 nm for pH 3 and pH 7.5, respectively. This was because large amounts of flocculation occurred, causing particle sedimentation and triggering phase separation. This electrostatically induced phase separation of mixtures of oppositely charged polyelectrolytes into a polymer-rich sediment and a coexisting dilute phase is a typical coacervation process.^46^ Although DLS was unable to detect these sediments, NPs coated with bPEI stabilized by the positive charge were still detected in the dilute phase, with good-quality data reported by DLS.

At pH 9, the measured ζ-potential value increased gradually from MR 0 to 2.0, and adsorption of bPEI reversed the charge at an MR of 1.3 (Figure 2a, green data). At pH 9, the PMES_500_ cores are fully ionized and bPEI had a low degree of protonation, with a large amount of abundant uncharged amine groups (Figure S4). In this situation, more bPEI adsorption onto NPs was required to neutralize the negative charges and particle coalescence occurred with MRs > 1.0 (Figure 2b, green data). When the MR was higher than 1.3, large particle aggregates were detected by DLS and sediment was not observed. In this situation, the NPs possibly acted as nanosized cross-linkers and bPEI as flexible bridges between adjacent NPs.

At pH 11, the measured ζ-potential value increased more gradually from MR 0 to 2.0 than that at pH 9 because bPEI had relatively low charge density at this pH (Figure 2a). Charge reversal did not occur between MRs 0 to 2.0 at pH 11, and the hydrodynamic diameter remained at approximately 300 nm for all mixtures tested at pH 11 (Figure 2b, pink data). In addition, sediment was also not observed.

DLS studies on these bPEI/NP mixtures at low concentration indicated that phase separation would occur for high-concentration mixtures at pH 3 and pH 7.5, and higher concentrations of bPEI would be required to trigger phase separation at the higher pH. However, based on this data, it was hypothesized that macroscopic gelation would occur at pH 9 and pH 11.

### bPEI/NP PNCC Formation

Aqueous PKSPMA_58_-PMES_500_ NP dispersions (20% w/w) and bPEI solutions (20% w/w) with the same pH were mixed at different bPEI-to-NPs MRs to observe the coacervation behavior of the bPEI/NP mixtures at high concentrations. In all cases herein, the pH measured after mixing remained unchanged.

At pH 3, the 20% w/w PKSPMA_58_-PMES_500_ NP dispersion was a milky liquid with a low viscosity (Figure S5a). On the addition of a small amount of bPEI (20% w/w, pH 3), flocculation was observed, and then phase separation occurred (Figure S5b). This observation is similar to that of the bPEI/PKSPMA_68_-PBzMA_300_ (5.6–0.1) mixture in our previous study. In this previous case, these NPs had a fully hydrophobic core with no ionizable functional groups. Similarly, at pH 3, PKSPMA_58_-PMES_500_ NPs had an uncharged, nonswollen, hydrophobic PMES core with an anionic sulfonate shell. The sulfonate shells of NPs were easily compensated by the highly protonated bPEI, leading to the loss of colloidal stability and thus causing bulk flocculation of the mixture.

Upon increasing the pH from 3 to 7.5, a fluid-to-gel transition occurred for the 20% w/w PKSPMA_58_-PMES_500_ NP dispersion and a free-standing hydrogel was obtained at pH 7.5, as judged by tube inversion tests (Figure S6a). This indicates that for these nanogels (swollen diameter = 440 nm), this concentration (20% w/w) is sufficient to be above the critical gel concentration.^47^ Initially, on the addition of bPEI (20% w/w, pH 7.5, MR < 0.2), the mixture remained as a free-standing hydrogel (Figure S6b) and became stiffer with stirring. This is likely because the colloidally stable, swollen nanogels adsorbed a layer of bPEI and were still able to absorb a relatively large amount of water. When excess bPEI was added (MR ≥ 0.2), the nanogels were unable to disperse evenly in the aqueous mixture and aggregated to form compact flocs, and the mixture began to flow (Figure S6c). When more bPEI was added (MR ≥ 0.4), phase separation occurred (Figure S6d) over a few seconds, resulting in a dense phase containing a relatively large content of polymer at the bottom of the vial, and a dilute aqueous phase exuded out from the dense phase.

Similar to the observations made at pH 7.5, the 20% w/w PKSPMA_58_-PMES_500_ NP dispersion at pH 9 formed a free-standing hydrogel (Figure S7a). Initially, on the addition of bPEI (20% w/w, pH 9, MR < 0.8), the bPEI/NP mixture remained as a hydrogel which did not flow due to gravity (Figure S7b) and became stiffer with stirring. As the amount of bPEI was further increased (MR ≥ 0.8), instead of phase separation, the mixture became a homogeneous fluid (Figure S7c). This observation was likely because the surface of the PKSPMA_58_-PMES_500_ NPs was totally covered by the adsorbed bPEI, which reversed the charge of the NPs, as shown in Figure 2a. After standing for 10 min, the bPEI/NP (9–1.0) fluid transformed into a free-standing hydrogel again, as judged by tube inversion tests (Figure S7d). With larger amounts of bPEI added, the bPEI/NP fluid required more time to transform into a free-standing hydrogel, e.g., 1 h for the formation of a bPEI/NP (9–1.9) PNCC hydrogel.

At pH 11, the 20% w/w PKSPMA_58_-PMES_500_ NP dispersion formed a free-standing hydrogel because of the swollen nanogels (Figure S8a). However, with the addition of bPEI (20% w/w, pH 11, MR ≤ 0.2), the mixture began to flow at first, but regelation occurred after 24 h, and the mixture bPEI/NP (11–0.2) turned into a yellow-colored hydrogel (Figure S8b). It is not fully understood why this color arose but was possibly caused by the inherent coloration of bPEI at this pH.

**Figure 3:**
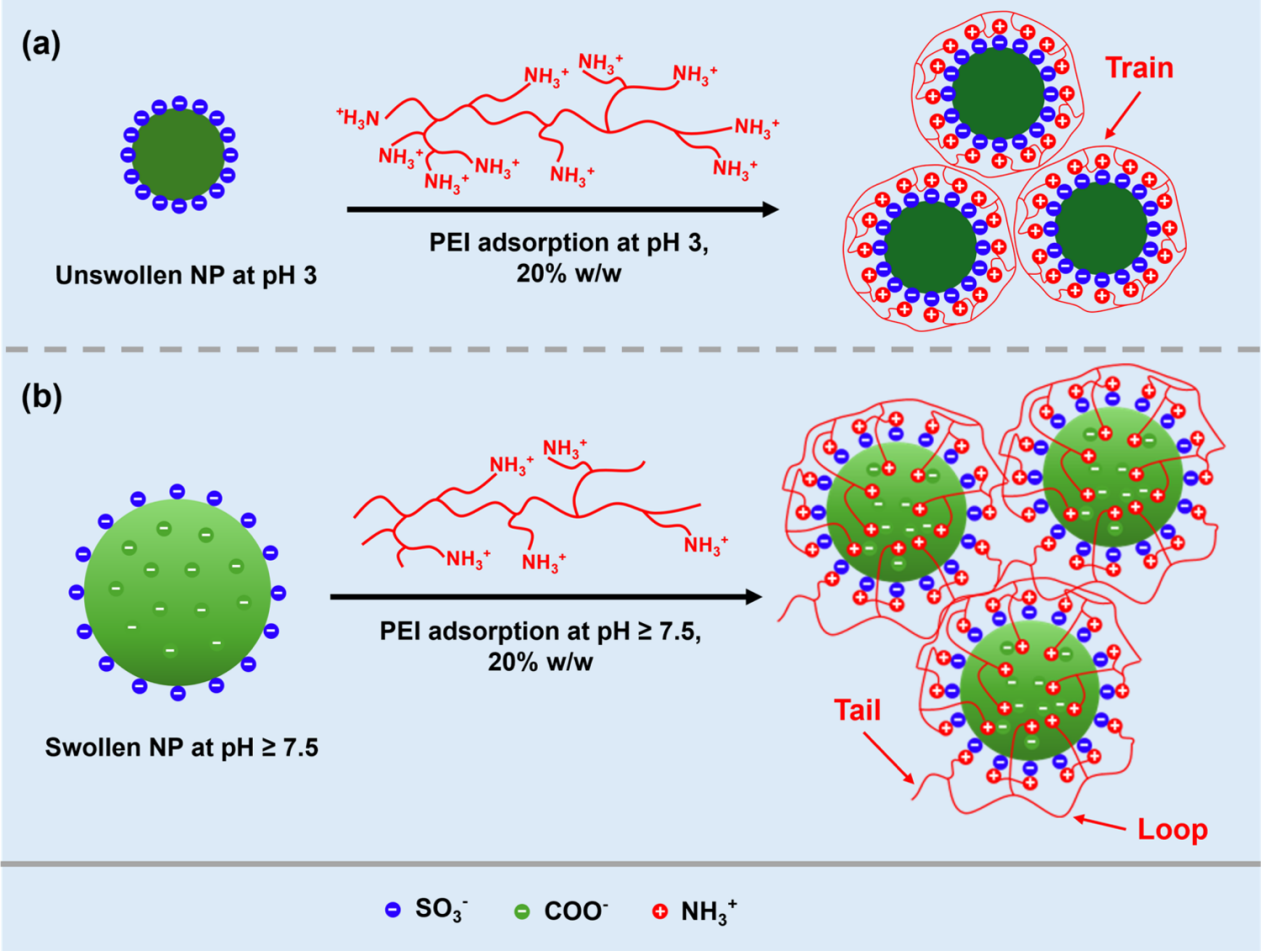
Different conformations of bPEI adsorption onto the PKSPMA_58_-PMES_500_ NP surfaces. Formation of (a) train conformation when NPs are unswollen and bPEI has a high charge density at pH 3 and (b) loop and tail conformations when NPs are swollen and bPEI has a low charge density at pH ≥ 7.5.

It is clear that there is an optimal bPEI-to-nanogel mass ratio for forming PNCC hydrogels at a given pH with the PNCC hydrogel preparation route. In addition, these PNCC hydrogels remain stable for at least 6 months (as judged by tube inversion tests), with related samples in our laboratory^38^ being stable for over one year. The different behaviors observed for bPEI/PKSPMA_58_-PMES_500_ mixtures prepared at varying MR and pH can be attributed to the conformation of adsorbed bPEI on the nanogel surface, as well as the status of the core of the nanogel. The adsorption of bPEI onto PKSPMA_58_-PMES_500_ nanogels was expected to occur through electrostatic interactions between the positive charge from bPEI and the negative charge from PKSPMA_58_-PMES_500_ nanogels. Both bPEI and PMES are weak polyelectrolytes, and therefore the charge density on these polymers varies with pH, resulting in strongly pH-dependent interactions.^48,49^ Three possible conformations of bPEI adsorption (train, loop, and tail) onto PKSPMA_58_-PMES_500_ NP surfaces, depending on solution pH, are illustrated in Figure 3.

At a low pH, bPEI molecules have high charge density and form relatively flat conformations (trains) on anionic surfaces (Figure 3a).^48^ Therefore, at pH 3, PKSPMA_58_-PMES_500_ nanoparticles have a nonswollen hydrophobic core and bPEI may adsorb onto the nanoparticles in train conformations. If the amount of cationic charge from bPEI is enough to compensate for the anionic charge from sulfonate groups on the nanoparticle surfaces, the nanoparticles aggregate and generate sediments. At a high pH, bPEI molecules have low charge density and tend to form “loop” and “tail” conformations (Figure 3b).^48^ At pH 7.5, 9, and 11, PKSPMA_58_-PMES_500_ nanogels have an ionized, swollen PMES core, and bPEI could adsorb onto the nanogels in loop and tail conformations. At these pHs, bPEI could interact with nanogels via ionic bonds between sulfonate groups from the PKSPMA block and the carboxylic acid groups from the PMES core. On the addition of bPEI, the nanogels shrink initially (Figure 2b), which is likely due to the complexation of carboxylic acid groups with amine groups. The amount of bPEI molecules capable of adsorbing onto the nanogels increased with increasing pH. At pH 7.5, the adsorbed bPEI molecules are not sufficient to stabilize the nanogels; thus, they aggregate and generate sediments. However, at pH 9 and pH 11, a large amount of bPEI can adsorb onto the nanogels in loop and tail conformations, which possibly generates steric hindrance via a large amount of abundant unprotonated bPEI chains. Therefore, at pH 9 and 11, the bPEI/NP mixtures remained homogeneous across a wide MR range (Figures S7 and S8). In addition, interparticle bridges could be generated and thus result in large particle clusters (Figure 2b).^45^ Related morphologies have been reported previously.^21,29,50^

### Rheology Studies on bPEI/NP Complex Coacervate Hydrogels

**Figure 4:**
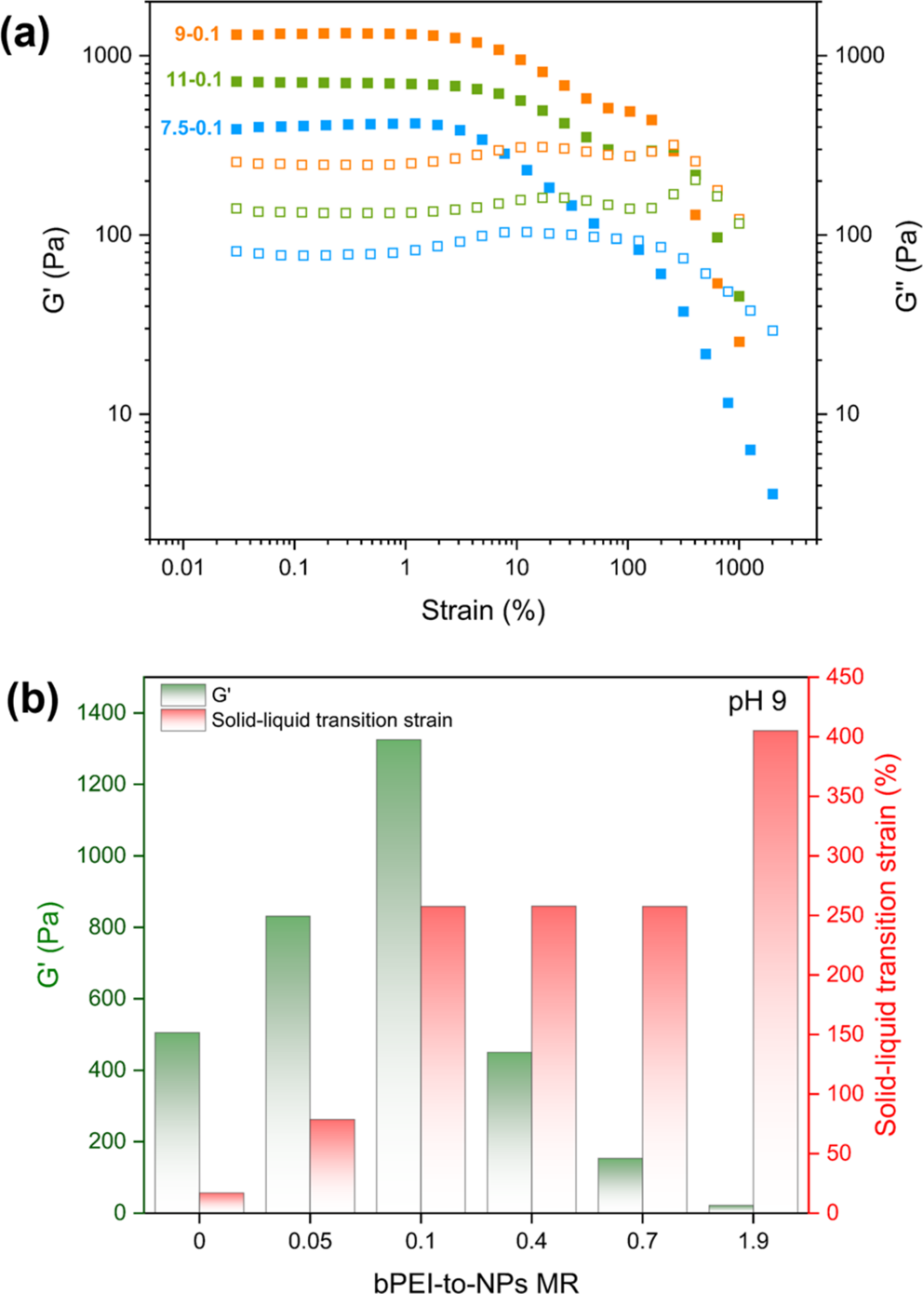
(a) Storage modulus (*G*′, solid squares) and loss modulus (*G*″, hollow squares) versus % strain of bPEI/NP complex coacervate hydrogels prepared at pH 7.5, pH 9, pH 11, with a fixed MR at 0.1 and (b) *G*′ (green) and the solid–liquid transition strain (red) for bPEI/NP complex coacervate hydrogels prepared at pH 9 with MRs from 0 to 1.9. Data was obtained by strain-dependent (ω = 10 rad s^–1^, 25 °C) oscillatory shear rheology.

The rheological properties of the bPEI/NP complex coacervate hydrogels were subsequently investigated by using oscillatory rheology. The measured storage modulus (*G*′) and loss modulus (*G*″) varied as the pH and MR were varied, as shown in Figures 4 and S9. Gelation occurred at MR 0.1 for all samples prepared at pH 7.5, pH 9, and pH 11, with measured gel strengths of 390, 1300, and 720 Pa, respectively (Figure 4a). The highest gel strength was observed at pH 9 and, as illustrated in Figure S7, gelation for bPEI/NP (20% w/w, pH 9) occurred over a wide MR range. As shown in Figure 4b, at this pH, the measured gel strengths (*G*′) varied as the MR was increased from 480 Pa (MR = 0) to 1300 Pa (MR = 0.1, Figure 4b). On increasing the MR from 0.1 to 1.9, the measured gel strength decreased to 20 Pa. In addition, with the addition of bPEI, the formed PNCC hydrogels became more stretchable and had solid–liquid transition points (the crossover point of *G*′ and *G*′′) of 250% (1.9 > MR ≥ 0.1), compared to a value of 145% for pristine swollen PKSPMA_58_-PMES_500_ nanogels. Interestingly, there is a double yield observed for PNCC hydrogels prepared at pH 9 (Figure S9) and pH 11 (Figure 4a), which is not yet fully understood.

**Figure 5:**
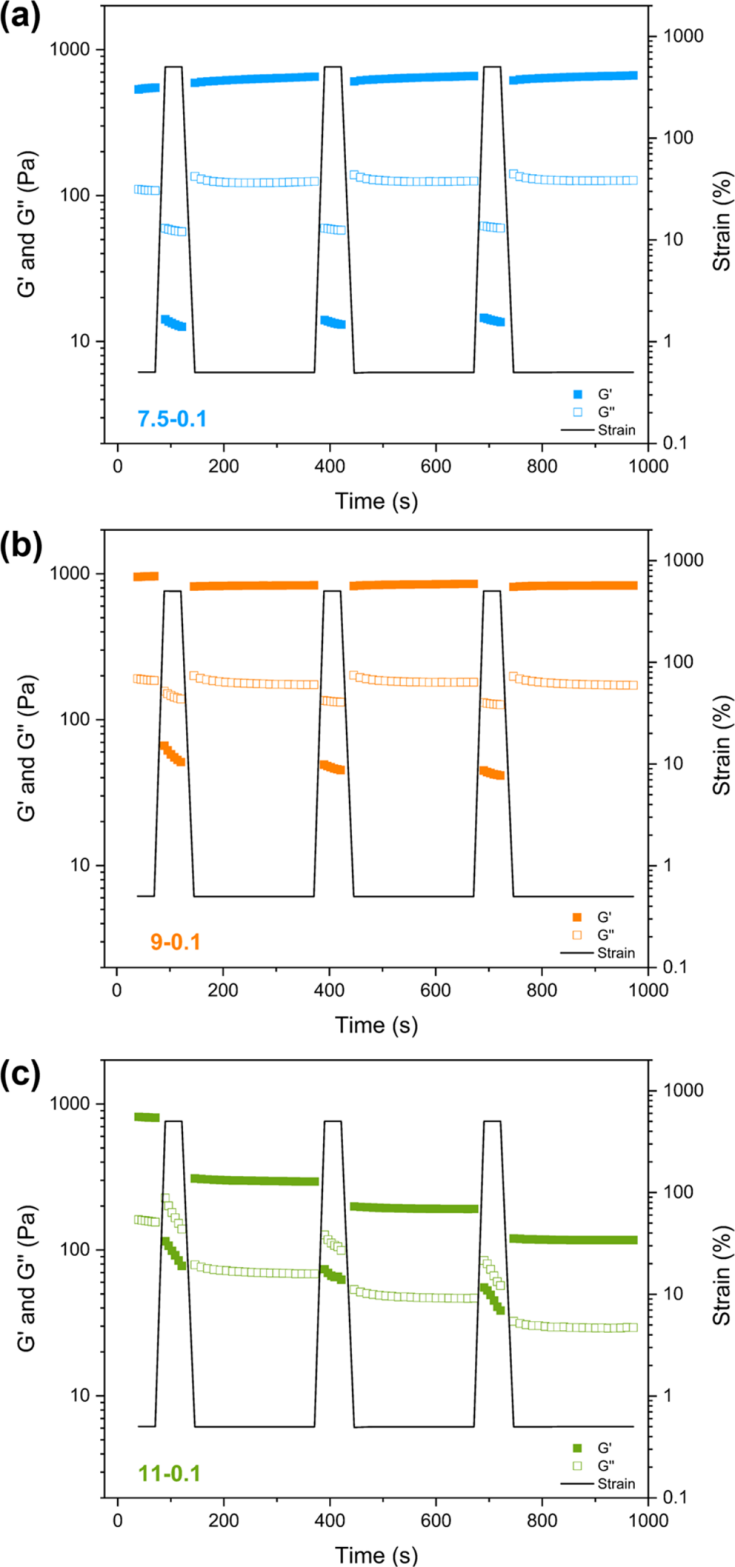
Oscillatory rheology recovery experiments (ω = 10 rad s^–1^, 25 °C) for (a) bPEI/NP (7.5–0.1), (b) bPEI/NP (9–0.1), and (c) bPEI/NP (11–0.1) complex coacervate hydrogels measured using a continuous strain sweep with alternating strain (ε = 0.5 and 500%).

The shear-thinning and recovery behavior of the bPEI/NP complex coacervate hydrogels were investigated by oscillatory rheology experiments, whereby the shear strain was varied between 0.5 and 500% (Figure 5). At pH 7.5, pH 9, and pH 11, when the initial shear strain was 0.5%, the coacervate hydrogels were within the linear viscoelastic region and showed solid-like behavior (*G*′ > *G*″). When the shear strain increased to 500%, the coacervate hydrogels shear thinned and had liquid-like behavior (*G*″ > *G*′). After removal of the high shear strain, bPEI/NP (7.5–0.1), bPEI/NP (9–0.1), and bPEI/NP (11–0.1) hydrogels recovered almost immediately to a solid-like state, indicating that these materials would serve as effective injectable gels for direct-ink writing printing.^38,51,52^

Although the gel strength of the bPEI/NP (11–0.1) hydrogel decreased upon each recovery cycle (Figure 5c), the bPEI/NP (7.5–0.1) hydrogel was able to recover 100% of the original gel strength (Figure 5a) and the bPEI/NP (9–0.1) hydrogel had a constant recovery efficiency at 87% (Figure 5b). The different recovery efficiency is possibly related to the different gelation rates observed for these hydrogels, where the gelation of bPEI/(NP) (11–0.1) required a longer time. In summary, these PNCC hydrogels exhibit tunable rheological properties based on formulation and pH.

## Conclusions

PNCC hydrogels were synthesized via mixing cationic bPEI and anionic PKSPMA_58_-PMES_500_ nanoparticles at 20% w/w. Gelation of bPEI/NP mixtures occurred at pH 7.5, pH 9, and pH 11 but was not observed at pH 3. Therefore, sulfonate-functional nanogels containing PMES nanoparticle cores need to be in their swollen state for bPEI/PKSPMA_58_-PMES_500_ complex coacervate hydrogel formation. DLS studies were useful in predicting the behavior of this bPEI/PISA-derived PKSPMA_58_-PMES_500_ nanogel system, which indicated that phase separation would occur at pH 3 and pH 7.5. Electrophoresis indicated the expected adsorption of bPEI onto these nanogels with more bPEI required to trigger charge reversal on the particle surface at higher pH.

All of the bPEI/NP complex coacervate hydrogels prepared had an improved solid-to-liquid transition point in comparison to PKSPMA_58_-PMES_500_ hydrogels formed from 20% w/w nanogels alone in their swollen form. In addition, mixing bPEI with PISA-derived nanogels yields PNCC hydrogels with increased yield points and gel strengths by controlling the mixture composition and pH. The maximum measured gel strength of the bPEI/PKSPMA_58_-PMES_500_ series was bPEI/NP (9–0.1), at approximately 1300 Pa. Shear-thinning behavior was observed for these PNCC hydrogels. The bPEI/NP (7.5–0.1) hydrogel and bPEI/NP (9–0.1) hydrogel exhibited 100 and 87% recovery efficiency, respectively, while the strength of the bPEI/NP (11–0.1) hydrogel decreased after each shear-thinning cycle.

## Supporting Information Available

Additional experimental materials and methods; ^1^H NMR spectra and GPC chromatogram of PKSPMA_58_ macro-CTA; pH values of bPEI solution diluted by HCl; degree of ionization of bPEI and nanoparticles at different pHs; additional digital photographs; and rheology data of bPEI/NPs mixtures prepared at pH 9 (PDF)
