# Supplementary material for: Investigating the Formation of Polymer–Nanoparticle Complex Coacervate Hydrogels Using Polymerization-Induced Self-Assembly-Derived Nanogels with a Succinate-Functional Core
Source: Langmuir. 2024 Sep 18;40(39):20648–56. doi: 10.1021/acs.langmuir.4c02626 (PMC11447913; doi:10.1021/acs.langmuir.4c02626)
Supplement: Supplementary file 1 — la4c02626_si_001.pdf [file la4c02626_si_001.pdf]

Supporting Information

# Investigating the formation of polymer nanoparticle complex coacervate hydrogels using polymerization-induced self-assembly-derived nanogels with a succinate-functional core

Ruiling Du,[1,2] Xueyuan Li,[1,2] and Lee A. Fielding[1,2*]

[1] Department of Materials, School of Natural Sciences, University of Manchester, Oxford Road, Manchester, M13 9PL, U.K.

[2] Henry Royce Institute, The University of Manchester, Oxford Road, Manchester, M13 9PL, U.K.

* lee.fielding@manchester.ac.uk

Number of pages: 9

Number of figures: 9

Number of schemes: 0

Number of tables: 0

**Table of Contents**

**Additional experimental materials and methods** ........ S3

**Figure S1.** (a) Synthesis of $PKSPMA_{58}$ macromolecular chain-transfer agent (macro-CTA) via RAFT solution polymerization. (b) Assigned $^1H$ NMR spectra of purified and freeze-dried $PKSPMA_{58}$ macro-CTA. The sample was dissolved in $D_2O$ prior to analysis. The DP for the purified polymer was calculated by comparing the integrated proton signals corresponding to the methacrylic polymer backbone at 2.83- 3.26 ppm and 3.97-4.44 ppm with that corresponding to the aromatic protons of the chain end at 7.2-7.4 ppm. ........ S5

**Figure S2.** Aqueous gel permeation chromatography chromatogram obtained for $PKSPMA_{58}$ macro-CTA. A relatively narrow molecular weight distribution was achieved, suggesting successful RAFT polymerization. ........ S5

**Figure S3.** 20 wt.% bPEI solutions ($M_n$ 60 kDa) pH values obtained by diluting bPEI (50 wt.%, 4 g) to 20 wt.% using different concentrations of HCl (6 g). ........ S6

**Figure S4.** Degree of protonation (blue) for 20% w/w bPEI solutions,[4] and degree of ionization (red) for 20% w/w $PKSPMA_{58}$-$PMES_{500}$ dispersions as a function of pH. ........ S6

**Figure S5.** Photographs of 20% w/w (a) $PKSPMA_{58}$-$PMES_{500}$ NP dispersion at pH 3 and (b) bPEI/NP (3-0.04). ........ S7

**Figure S6.** Photographs of 20% w/w (a) $PKSPMA_{58}$-$PMES_{500}$ NP dispersion at pH 7.5, (b) bPEI/NP (7.5-0.1), (c) bPEI/NP (7.5-0.2), and (d) bPEI/NP (7.5-0.4). ........ S7

**Figure S7.** Photographs of 20% w/w (a) $PKSPMA_{58}$-$PMES_{500}$ NP dispersion at pH 9, (b) bPEI/NP (9-0.25), (c) bPEI/NP (9-0.8), and (d) bPEI/NP (9-1.0). (e) Gelation of 20% w/w bPEI/NP (9-1.0) after 10 min. ........ S8

**Figure S8.** Photographs of 20% w/w (a) $PKSPMA_{58}$-$PMES_{500}$ NP dispersion at pH 9, and (b) bPEI/NP (11-0.2). ........ S8

**Figure S9.** Storage modulus (G', solid squares) and loss modulus (G'', hollow squares) versus % strain of bPEI/NP complex coacervate hydrogels prepared at pH 9 with MR from 0 to 1.9. Data is obtained by strain-dependent ($\omega$ = 10 rad $s^{-1}$, 25 °C) oscillatory shear rheology. ........ S9

**References** ........ S9

## Additional experimental materials and methods

**Materials.** 3-sulfopropyl methacrylate potassium salt (KSPMA, 98 %), mono-2-(methacryloyloxy)ethyl succinate (MES, 95 %, inhibited with 500 ppm MEHQ) and 4,4′-azobis(4-cyanovaleric acid) (ACVA, 98 %) were purchased from Sigma-Aldrich (UK) and used as received. Methanol and 1,4-dioxane were purchased from Sigma-Aldrich (UK) and were organic synthesis grade. 4-Cyano-4-(2-phenylethane sulfanylthiocarbonyl) sulfanylpentanoic acid (PETTC) was synthesized in-house using previously reported protocols.[1] Deuterated NMR solvent ($D_2O$) for NMR characterization was purchased from Cambridge Isotope Laboratories (UK). Dialysis tubing (regenerated cellulose, MWCO = 3.5 kDa, diameter = 29 mm) was received from Fisher Scientific (UK).

**Synthesis of $PKSPMA_{58}$ via RAFT solution polymerization.** The preparation of PKSPMA by RAFT solution polymerization has been reported previously.[1-3] The protocol used for the preparation of $PKSPMA_{58}$ is as follows (Figure S1a). KSPMA monomer (18.0 g, 73.1 mmol), PETTC RAFT agent (427.8 mg, 1.3 mmol, dissolved in dioxane), ACVA (70.6 mg, 0.3 mmol, PETTC/ACVA molar ratio = 5), and pH 5.5 acetate buffer (78.3 g, final buffer/dioxane ratio = 3) were weighed into a 250 ml round-bottomed flask, which was then sealed and purged with $N_2$ for 30 min. The sealed flask was placed in a preheated water bath at 70 °C for 90 min. The reaction was quenched by immersion in an ice bath and opening the flask to air. The resulting PKSPMA was purified by dialysis against 10:1 water/methanol overnight. The purified solution was dried under vacuum at 30 °C to evaporate volatiles and then freeze-dried from aqueous solution overnight to yield a yellow product (65.3 % yield). The mean degree of polymerization (DP) of PKSPMA was calculated to be 58, as determined by $^1H$ NMR using $D_2O$ (Figure S1b). $PKSPMA_{58}$ macro-CTA had a molar mass dispersity ($M_w/M_n$) of 1.12 (Figure S2), which is consistent with previous studies reporting well-controlled RAFT syntheses.[1]

**Preparation of $PKSPMA_{58}$-$PMES_{500}$ diblock copolymer nanoparticles via RAFT aqueous emulsion polymerization.** The preparation of PKSPMA-PMES by RAFT aqueous emulsion polymerization has been reported before.[3] A typical polymerization using a MES : $PKSPMA_{58}$ : ACVA ratio of 500 : 1: 0.25 at 20 % w/w solids at pH 2 water is as follows. $PKSPMA_{58}$ (0.4 g, 0.037 mmol), MES (4.303 g, 18.692 mmol), and ACVA (2.6 mg, 9.35 μmol; CTA/initiator molar ratio = 4.0) were weighed into a 50 mL round bottomed flask. Deionized water and 0.25 M HCl were added to adjust the mixture to pH 2.5 to prevent ionization of the -COOH groups on MES during the polymerization and to produce a 20% w/w aqueous mixture. The solution was then purged with $N_2$ for 30 min prior to immersion in a water bath set at 70 °C. The heated reaction was stirred for 24 h before the polymerization was quenched by cooling in an ice bath and exposure to air.

**Proton nuclear magnetic resonance ($^1$H NMR)** spectra were recorded on a Bruker Advance III 400 MHz spectrometer with 128 scans averaged per spectrum at 25 °C.

**Aqueous gel permeation chromatography (GPC)** measurements were carried out using a phosphate buffer eluent (pH 9) containing 30 % v/v methanol at a flow rate of 1.0 mL min$^{-1}$ at ambient temperature. The instrument was fitted with two PL aquagel-OH MIXED-H 8 μm columns and a refractive index detector (Shodex RI-101) was used to determine molar mass distributions. The system was calibrated with a series of near-monodisperse poly(ethylene oxide) standards. Samples were prepared at a concentration of 2 mg mL$^{-1}$ in the phosphate buffer eluent.

## Supporting Figures

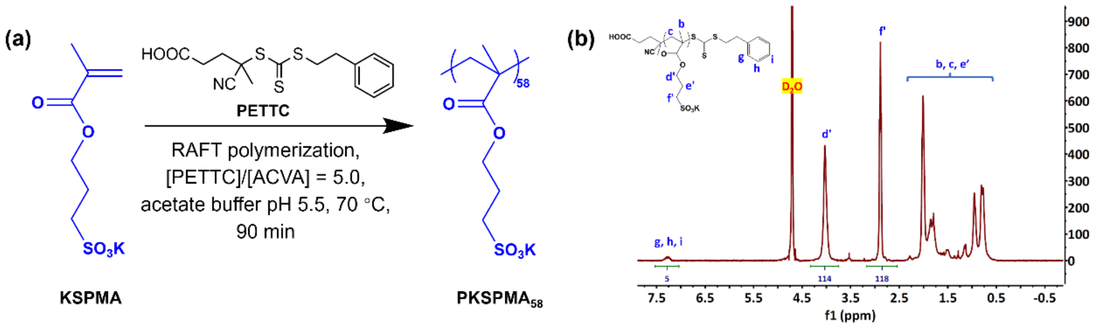


**Figure S1.** (a) Synthesis of $PKSPMA_{58}$ macromolecular chain-transfer agent (macro-CTA) via RAFT solution polymerization. (b) Assigned $^{1}H$ NMR spectra of purified and freeze-dried $PKSPMA_{58}$ macro-CTA. The sample was dissolved in $D_2O$ prior to analysis. The DP for the purified polymer was calculated by comparing the integrated proton signals corresponding to the methacrylic polymer backbone at 2.83-3.26 ppm and 3.97-4.44 ppm with that corresponding to the aromatic protons of the chain end at 7.2-7.4 ppm.

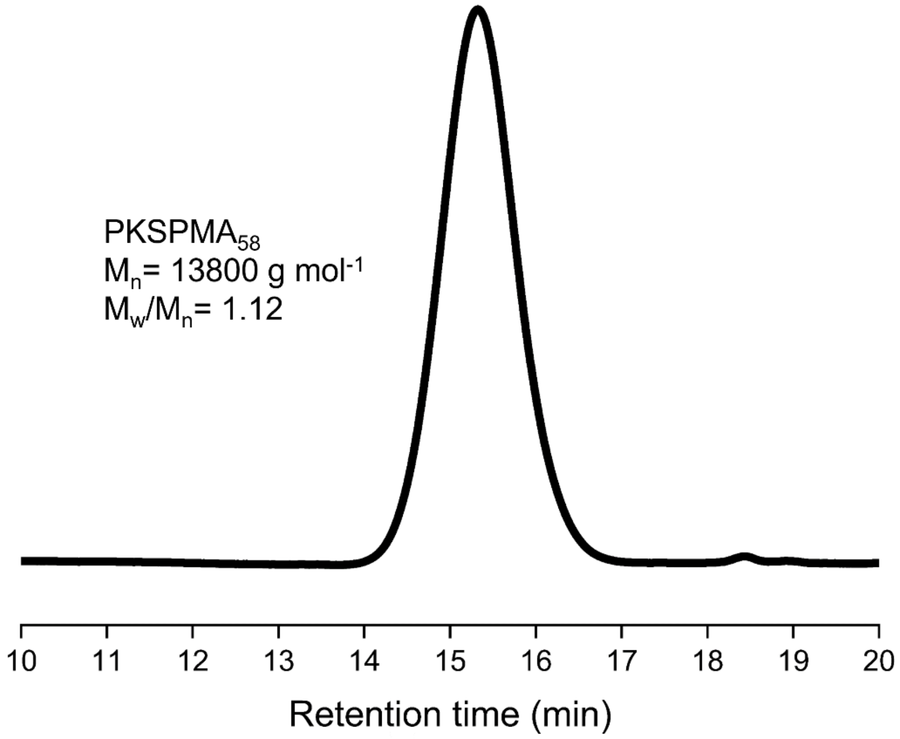


**Figure S2.** Aqueous gel permeation chromatography chromatogram obtained for $PKSPMA_{58}$ macro-CTA. A relatively narrow molecular weight distribution was achieved, suggesting successful RAFT polymerization.

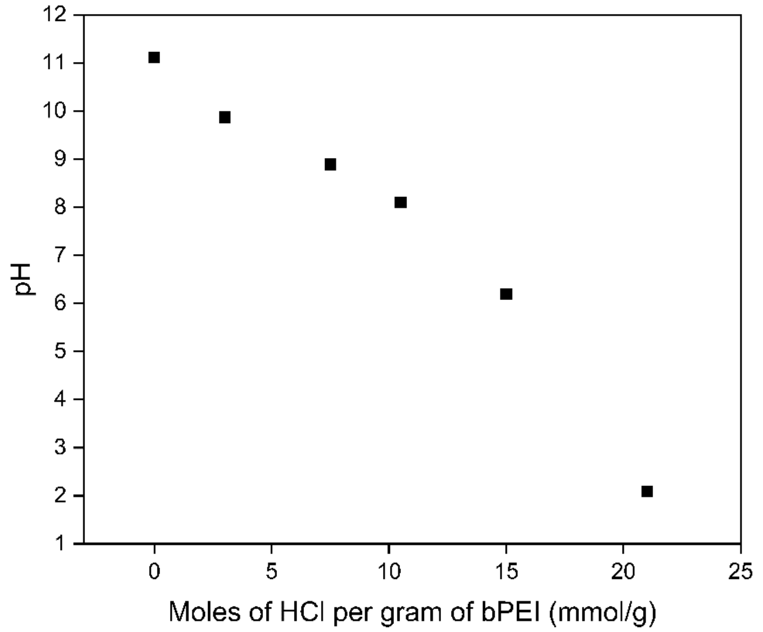


**Figure S3.** 20 wt.% bPEI solutions ($M_n$ 60 kDa) pH values obtained by diluting bPEI (50 wt.%, 4 g) to 20 wt.% using different concentrations of HCl (6 g). Moles of HCl per gram of bPEI was calculated using the following equation: $Moles\ of\ HCl\ per\ gram\ of\ bPEI = \frac{Concentration\ of\ HCl \times Mass\ of\ HCl\ solution}{Concentration\ of\ bPEI \times Mass\ of\ bPEI\ solution}$

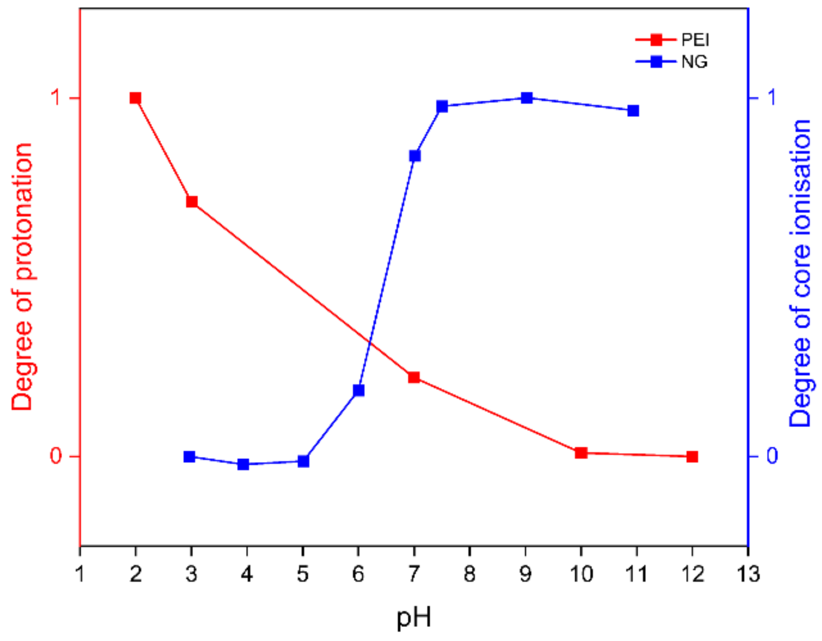


**Figure S4.** Degree of protonation (blue) for 20% w/w bPEI solutions,[4] and degree of ionization (red) for 20% w/w PKSPMA$_{58}$-PMES$_{500}$ dispersions as a function of pH.

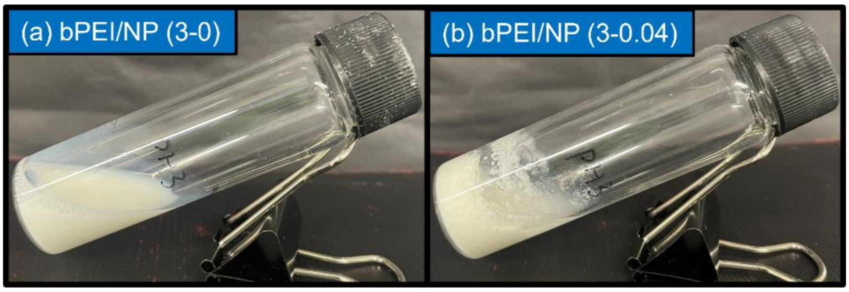


**Figure S5.** Photographs of 20% w/w (a) $PKSPMA_{58}$-$PMES_{500}$ NP dispersion at pH 3 and (b) bPEI/NP (3-0.04).

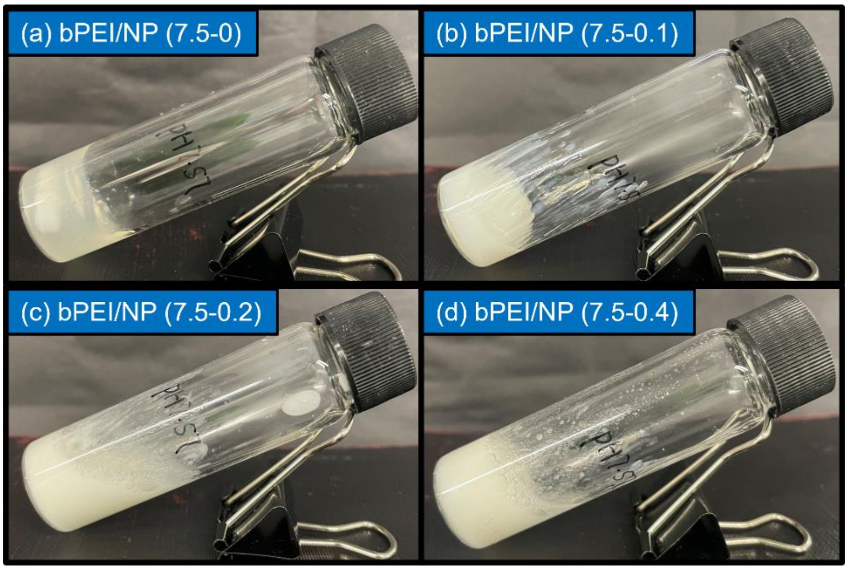


**Figure S6.** Photographs of 20% w/w (a) $PKSPMA_{58}$-$PMES_{500}$ NP dispersion at pH 7.5, (b) bPEI/NP (7.5-0.1), (c) bPEI/NP (7.5-0.2), and (d) bPEI/NP (7.5-0.4).

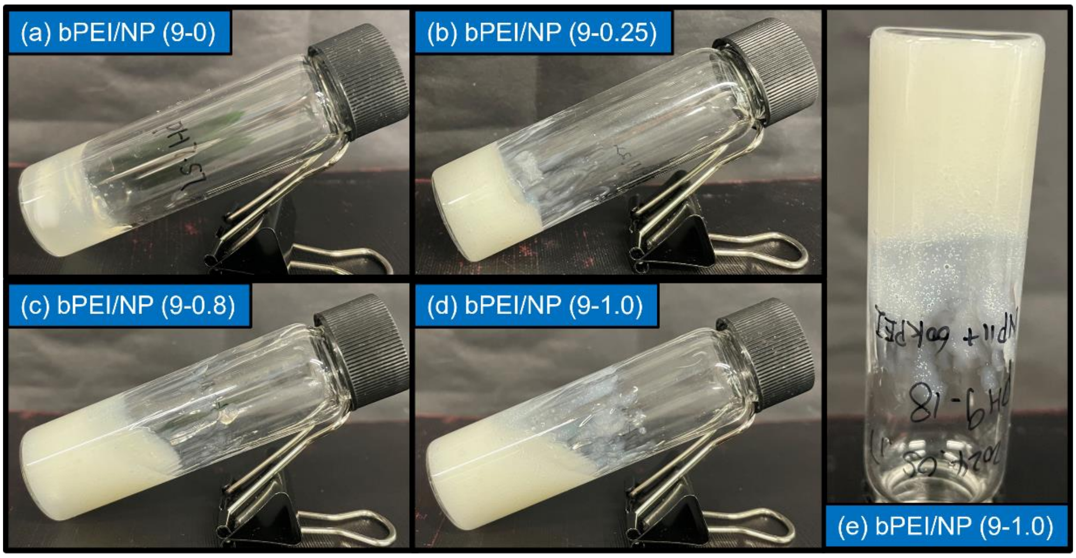


**Figure S7.** Photographs of 20% w/w (a) PKSPMA$_{58}$-PMES$_{500}$ NP dispersion at pH 9, (b) bPEI/NP (9-0.25), (c) bPEI/NP (9-0.8), and (d) bPEI/NP (9-1.0). (e) Gelation of 20% w/w bPEI/NP (9-1.0) after 10 min.

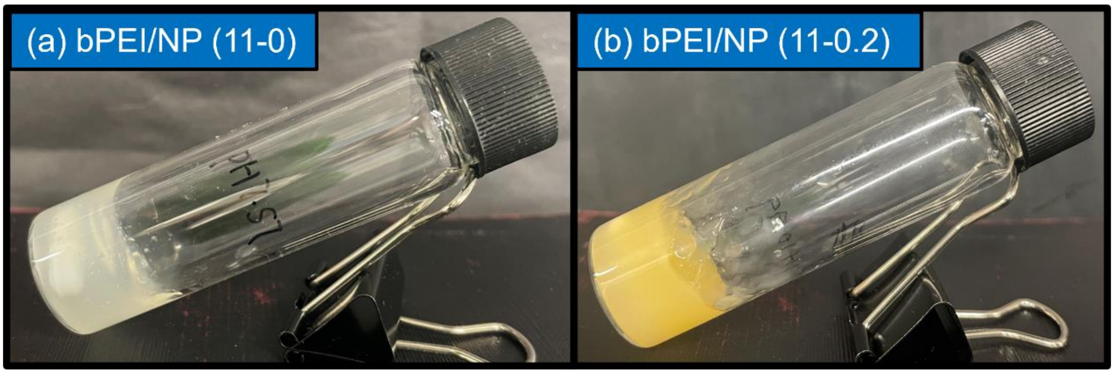


**Figure S8.** Photographs of 20% w/w (a) PKSPMA$_{58}$-PMES$_{500}$ NP dispersion at pH 9, and (b) bPEI/NP (11-0.2).

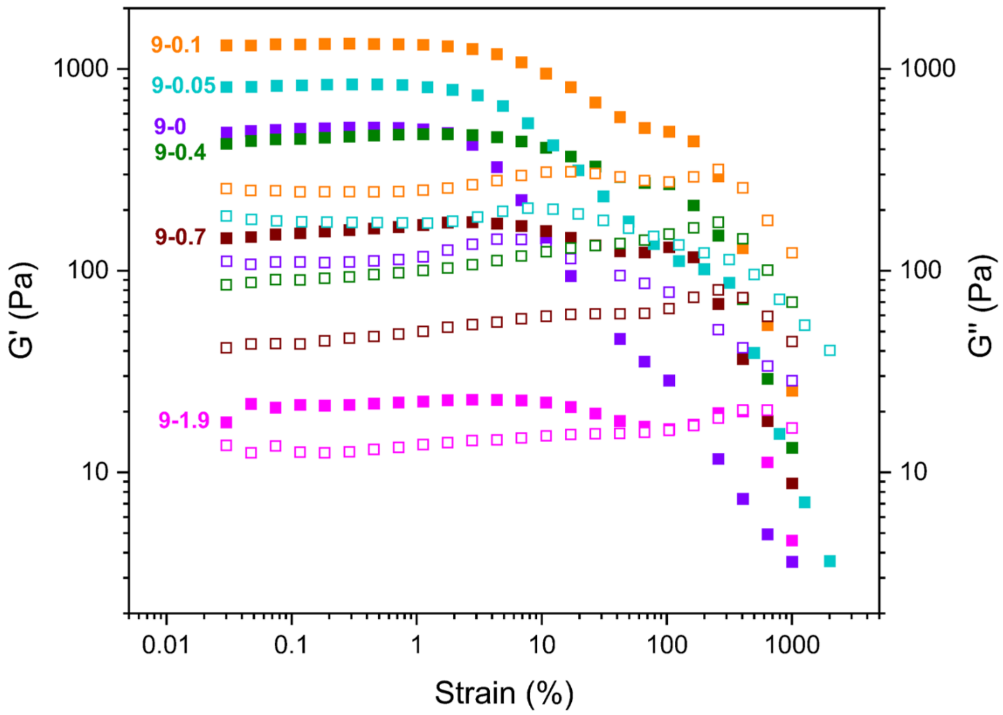


**Figure S9.** Storage modulus (G', solid squares) and loss modulus (G'', hollow squares) versus % strain of bPEI/NP complex coacervate hydrogels prepared at pH 9 with MR from 0 to 1.9. Data is obtained by strain-dependent ($\omega = 10$ rad $s^{-1}$, 25 °C) oscillatory shear rheology.

**References**

(1) Wen, S.-P.; Saunders, J. G.; Fielding, L. A. Investigating the influence of solvent quality on RAFT-mediated PISA of sulfonate-functional diblock copolymer nanoparticles. *Polymer Chemistry* **2020**, *11* (20), 3416-3426.

(2) Du, R.; Fielding, L. A. Preparation of polymer nanoparticle-based complex coacervate hydrogels using polymerisation-induced self-assembly derived nanogels. *Soft Matter* **2023**, *19* (11), 2074-2081.

(3) Du, R.; Fielding, L. A. pH-Responsive Nanogels Generated by Polymerization-Induced Self-Assembly of a Succinate-Functional Monomer. *Macromolecules* **2024**, *57* (8), 3496-3501.

(4) Wang, H.; Wang, Y.; Yan, H.; Zhang, J.; Thomas, R. K. Binding of sodium dodecyl sulfate with linear and branched polyethyleneimines in aqueous solution at different pH values. *Langmuir* **2006**, *22* (4), 1526-1533.
